# Supplementary material for: Screening People with Tuberculosis for High Risk of Severe Illness at Notification: Programmatic Experience from Karnataka, India
Source: Trop Med Infect Dis. 2021 Jun 15;6(2):102. doi: 10.3390/tropicalmed6020102 (PMC8293347; doi:10.3390/tropicalmed6020102)
Supplement: Supplementary file 1 [file tropicalmed-06-00102-s001.zip › tropicalmed-1230349-supplementary/Supplementary Files/Supplementary File S2.pdf]

# Data collection tool – Screening for high risk of severe illness at notification

This assessment should be done at public PHI for all ADULT (15 years or above) notified TB patients (not known to be drug resistant). Screening must be done as early as possible (at diagnosis or at the time of baseline investigations of TB patient at PHI). Store these filled forms at PHI. If high risk of severe illness is present please inform the STS at the earliest. This is being done on a pilot basis in select districts of Karnataka for patients notified between 15 October and 30 November 2020 (take help of M.O if required)

**VARIABLES**

**Nikshay ID: _________ State: Karnataka NTEP District: ______________ TU number: ____**

**Public Peripheral Health Institution (PHI) name: ____________________**

**Is the TB patient admitted in hospital at the time of screening (Tick one option)**

○ No ○ Yes

- Weight (kilograms (kg), max one decimal point) - ___________________
- Height (centimetre) – ___________________
- BMI (kilogram per metre square (kg/$m^2$), max one decimal point) - _________________
- Swelling in the leg (press for 30 sec inner side, 2cm above the ankle), tick only one option
  ○ No ○ Yes
- Respiratory rate per minute (in sitting position at rest) - ___________
- Oxygen saturation (%) using pulse oximeter (optional if available) measured at rest- ___________
- Able to stand without support (tick appropriate response), choose only one option
  ○ No (not able to stand without support) ○ Yes (standing without support)
- **High risk of severe illness,** tick only one option (see criterion on the back side of this page)
  ○ No ○ Yes

**Date of completing screening and this form at PHI (dd/mm/yyyy): _______________**

**Name and designation of staff completing the form: ________________________**

**FOR INFORMATION ONLY – Tool to screen 'high risk of severe illness'**

**If at least one of the following is present (any one), then the person with TB is 'high risk of severe illness'**

Body mass index (BMI) less than or equal to (≤) 14.0 kg/$m^2$ (OR)

BMI less than or equal to (≤) 16.0 kg/$m^2$ with leg swelling (OR)

Respiratory rate more than (>) 24 per minute (OR)

Oxygen saturation less than (<) 94% (OR)

Not able to stand without support (standing with support / squatting / sitting / bed ridden)
